# *In vitro* Susceptibility of Human Cell Lines Infection by Bovine Leukemia Virus

**DOI:** 10.3389/fmicb.2022.793348

**Published:** 2022-03-14

**Authors:** Nury N. Olaya-Galán, Skyler Blume, Kan Tong, HuaMin Shen, Maria F. Gutierrez, Gertrude C. Buehring

**Affiliations:** ^1^Ph.D. Program in Biomedical and Biological Sciences, School of Medicine and Human Health, Universidad del Rosario, Bogotá, Colombia; ^2^Grupo de Enfermedades Infecciosas, Laboratorio de Virología, Departamento de Microbiología, Pontificia Universidad Javeriana, Bogotá, Colombia; ^3^School of Public Health, University of California, Berkeley, Berkeley, CA, United States

**Keywords:** bovine leukemia virus, *in vitro* infection, human cell lines, zoonotic potential, cell-to-cell infection

## Abstract

Evidence of the presence of bovine leukemia virus (BLV) in human beings and its association with breast cancer has been published in the literature, proposing it as a zoonotic infection. However, not enough evidence exists about transmission pathways nor biological mechanisms in human beings. This study was aimed at gathering experimental evidence about susceptibility of human cell lines to BLV infection. Malignant and non-malignant human cell lines were co-cultured with BLV-infected FLK cells using a cell-to-cell model of infection. Infected human cell lines were harvested and cultured for 3 to 6 months to determine stability of infection. BLV detection was performed through liquid-phase PCR and visualized through *in situ* PCR. Seven out of nine cell lines were susceptible to BLV infection as determined by at least one positive liquid-phase PCR result in the 3-month culture period. iSLK and MCF7 cell lines were able to produce a stable infection throughout the 3-month period, with both cytoplasmic and/or nuclear BLV-DNA visualized by IS-PCR. Our results support experimental evidence of BLV infection in humans by demonstrating the susceptibility of human cells to BLV infection, supporting the hypothesis of a natural transmission from cattle to humans.

## Introduction

Viral agents have been linked to approximately 20% of human cancer types, and some causative relationships have been established (Morales-Sánchez and Fuentes-Pananá, 2014). The most common examples of those relationships include human papilloma virus (HPV) with cervical cancer, hepatitis B and C viruses (HBV, HCV) with liver cancer, Epstein–Barr virus (EBV) with Burkitt’s lymphoma, and human herpes virus 8 (HHV8) with Kaposi’s sarcoma. Breast cancer has long been studied as a possible candidate for a virus-caused human cancer due to the evidence of some viral markers present on breast cancer tissues (Lehrer and Rheinstein, 2019; Al Hamad et al., 2020; Lawson and Glenn, 2021). In the last decade, bovine leukemia virus (BLV) has been proposed as a possible risk factor for breast cancer development in different regions as a result of case–control studies in which statistically significant associations of the presence of the virus with breast cancer patients has been identified (Buehring et al., 2015; Lendez et al., 2018; Schwingel et al., 2019; Delarmelina et al., 2020; Olaya-Galán et al., 2021b).

Bovine leukemia virus is an exogenous retrovirus, grouped with human T-cell leukemia virus (HTLV) in the deltaretrovirus genus. These viruses cause leukemia/lymphoma both in cattle and humans, respectively (Lairmore, 2014). Cancer development could take more than 5–10 years post-infection to occur, although a low percentage (about 5–10%) of the infected population develops the last stages of the disease. BLV is distributed worldwide with prevalence rates between 10 and 90% in cattle, although North and South America have some of the highest prevalence rates (70–90%) (Polat et al., 2017). One of the biggest challenges of BLV infection is that most of the infected animals remain asymptomatic in the herds favoring the transmission and dissemination processes as no vaccine is available and diagnosis is not performed broadly (OIE, 2021).

Previous research has reported the presence of BLV biomarkers in humans, such as gene segments, viral proteins, and antibodies against BLV, which provides clear evidence of the presence of the virus in this host (Buehring et al., 2003, 2014, 2019; Ochoa Cruz et al., 2006; Mesa et al., 2013; Robinson et al., 2016; Khalilian et al., 2019). Studies of BLV in humans have been based on epidemiological analyses for the viral detection and association with breast cancer, but still there is a gap in the knowledge, viz. how does this virus (which naturally infects cattle) reach the human population and infect human beings. Therefore, concerns about the zoonotic potential of BLV have been present for some time in the BLV research, and for several years, researchers have tried to show the implications of BLV in human beings (Burridge, 1981; Burny et al., 1985; Buehring et al., 2003). In early BLV research, it was not possible to identify any relationship between humans and BLV (Burridge, 1981), but now there is increasing evidence about the presence of the virus in human beings, strengthening the hypothesis of BLV being a zoonotic agent (Cuesta et al., 2018; Buehring and Sans, 2020; Canova et al., 2021; Corredor-Figueroa et al., 2021).

Even if cattle is the natural host of the virus, evidence of the presence of BLV has also been reported in other species (Lee et al., 2012; Barez et al., 2015; Olaya-Galán et al., 2021a). Thus, BLV has been described as a versatile agent that could infect multiple hosts both naturally and under controlled conditions in the laboratory (Gillet et al., 2007). In cattle, the target cells of the virus are the B lymphocytes (Domenech et al., 2000), although in other studies *in vitro* infection has been evaluated in cell lines of different origins/sources including other bovine tissues, and some other animal species (Inabe et al., 1998; Domenech et al., 2000; Camargos et al., 2014; Iwan et al., 2014; Martinez Cuesta et al., 2018). However, few studies have been carried out regarding the infection of BLV in human cell lines and its implications of infection (Altaner et al., 1989; Tajima et al., 2003; Suzuki et al., 2018).

For BLV and its close relative HTLV, low amounts of free viral particles are released in the viral cycles compared with other retroviruses, and thus, a cell-to-cell transmission is needed to reach uninfected cells (Lairmore, 2014; Gross and Thoma-Kress, 2016). Although it is rare and less efficient, free viral particles could also be released from infected cells and perform a classic viral cycle of infection mediated by cellular receptors such as AP3D1 or CAT1/SLC7A1 proteins (Corredor et al., 2018; Bai et al., 2019). This research was focused on investigating if human cell lines from different tissues were susceptible of infection with the BLV under controlled conditions in the laboratory. Our results provide evidence supporting the hypothesis of the zoonotic potential of the virus and providing a model of human infection for further studies, as stable infection was reached in two different human cell lines.

## Materials and Methods

### Cell Lines and Culture Conditions

Fetal Lamb Kidney (FLK) cells, constitutively infected with BLV, served as a repository of the virus. Minimal Essential Medium (MEM) was used for cell passage every 3–4 days after 80% confluency. Human cell lines used and growing conditions are described in Table 1 (Pulvertaft, 1964; Soule et al., 1973, 1990; Pattillo et al., 1977; Peebles et al., 1978; Dexter et al., 1979; DuBridge et al., 1987; Roecklein and Torok-Storb, 1995; Stürzl et al., 2013). For each experiment, viability was verified by trypan blue stain and cells were counted through Neubauer’s chamber technique.

**TABLE 1:** Cell lines used in *in vitro* infection experiments and growth conditions.

Cell line	Cell/tissue type	Biological status	Medium + 10% FBS	Passage (days)
RaJi (ATCC CCL-86)	B cell—Lymphoblast	Burkitt’s lymphoma + EBV	RPMI-1640	2–3
HS-27 (ATCC CRL-1634)	Fibroblast—skin	Non-malignant	DMEM	7–8
MCF 102A (ATCC CRL-10781)	Epithelial—mammary gland	Non-malignant	DMEM/F12, 1:1	3–4
MCF 7 (ATCC HTB-22)	Epithelial—mammary gland	Adenocarcinoma	DMEM	4–5
CaSki (ATCC CRL-1550)	Epithelial—cervix	Epidermoid carcinoma + HPV	RPMI-1640	3–4
G361 (ATCC CRL-1424)	Epithelial—skin	Malignant melanoma	MEM	3–4
293T (ATCC CRL-3216)	Epithelial—embryonic kidney	Non-malignant + adenovirus	DMEM	2–3
DLD-I (ATCC CCL-221)	Epithelial—colon	Colorectal carcinoma	RPMI-1640	4–5
iSLK (UC Berkeley donation)	Epithelial—kidney	Renal carcinoma + latent KSHV	DMEM	4–5

### TransWell Infections

To recreate a plausible scenario of infection of BLV, TransWells (Costar Corning Inc.) with 0.4-μm polyester pore membrane were inserted above a 12-well plate to co-culture BLV-infected cells with human cell lines. The TransWell pores do not allow whole cells to pass through the pores but do allow extensions of cells (e.g., nanotubes or cellular conduits) to make contact below the insert, allowing a cell-to-cell transmission of the virus. Before infections, cell lines were verified to be negative to BLV (Supplementary Figure 1).

Uninfected human cell lines were cultured in the lower compartments with an approximate concentration of 10^5^ cells/ml until 70–80% confluency was reached in their respective media (Table 1). Thereafter, FLK cells were seeded in the TransWells (6,000 cells per well). For experimental infections, co-cultures were incubated for 48 h and then the human cell lines in the lower compartment were harvested. The BLV-infected human cell lines were scaled up into T25 cell culture flasks for follow-up of the infection, for up to 3–6 months to determine the stability of BLV infection.

As experimental controls, human cell lines with PBS 1X instead of FLK cells in the TransWell were also collected and maintained simultaneously with infected ones to compare morphology or any other visible change caused due to the viral infection. Infection experiments were repeated twice for the study, at two independent and different times. Manipulation of cell lines was carried out independently, reducing the risk of cross-contamination of cell cultures. Infections per cell line were performed on different days, and for the maintenance and follow-up, separate hoods for infected and non-infected cell lines were used as well. In addition, cell lines were initially validated to be negative to BLV prior infection with the PCRs used for viral detection (see next section).

### Bovine Leukemia Virus Detection and Follow-up of Infection

After 48 h post-infection (hpi) with the TransWells, an aliquot of the cells in the bottom plate was recovered to perform DNA extraction with the DNeasy Kit from QIAgen following the manufacturer’s instructions. Total DNA recovered was stored at −20°C until further use. Human GAPDH housekeeping gene was used as a validation control of the DNA extraction. Sheep *cytochrome C oxidase* housekeeping gene was used to verify that FLK cells did not trespass the membrane of the TransWells and human cell lines were not contaminated with FLK.

Viral genome was detected by PCR (see next section). Successful infected human cell lines were considered those in which BLV-DNA was detected in the initial DNA extraction post-infection and were maintained in cell culture with its respective conditions (Table 1). A follow up of the positive cell lines was performed every 2 weeks in which an aliquot of infected cells and controls without infection were taken for DNA extraction and PCR detection (Table 2).

**TABLE 2:** Evidence of BLV-GRE genomic region in human cell lines after infection through nPCR in a follow-up of 12 weeks post-infection.

Cell line	Replicate	Evidence of BLV post-infection				
		t0	Week 2	Week 4	Week 8	Week 12
RaJi (ATCC CCL-86)	1	Pos	Pos	Neg	Neg	Neg
	2	Pos	Pos	Neg	Neg	Neg
HS-27 (ATCC CRL-1634)	1	Pos	Pos	Pos	Pos	Neg
	2	Pos	Pos	Pos	Neg	Neg
MCF 102A (ATCC CRL-10781)	1	Pos	Pos	Pos	Neg	Neg
	2	Pos	Pos	Pos	Neg	Neg
MCF 7 (ATCC HTB-22)	1	Pos	Pos	Pos	Pos	Pos
	2	Pos	Pos	Pos	Neg	Neg
CaSki (ATCC CRL-1550)	1	Neg	–	–	–	–
	2	Neg	–	–	–	–
G361 (ATCC CRL-1424)	1	Neg	–	–	–	–
	2	Neg	–	–	–	–
293T (ATCC CRL-3216)	1	Pos	Neg	Neg	Neg	Neg
	2	Pos	Pos	Neg	Neg	Neg
DLD-I (ATCC CCL-221)	1	Pos	Pos	Pos	Neg	Neg
	2	Pos	Pos	Pos	Neg	Neg
iSLK	1	Pos	Pos	Pos	Pos	Pos
	2	Pos	Pos	Pos	Pos	Pos

Viral genes *LTR*, *gag*, *pol*, *env*, and *tax* were tested by liquid-phase nested PCR (nPCR) with GoTaq Promega to verify the presence of the complete genome of the virus at time 0 post-infection. Primers used for the viral detection and PCR conditions were used from previous studies (Buehring et al., 2014; Corredor et al., 2018). For the follow-up, detection of the GRE region of the LTR of the virus was used as a biomarker of viral infection, as it is one of the most conserved genes of the virus and belong to the 5′ limit of the viral genome. Results were visualized by electrophoresis in a 1.5% agarose gel, TBE1x, stained with ethidium bromide, and run conditions for 30 min—100 V in TBE1x. PCR conditions and primers are shown in the Supplementary Table 1.

For those cell lines that showed a stable infection of BLV during the total time of the follow-up, an *in situ* PCR (IS-PCR) was performed after 16 weeks of infection to confirm the presence of the virus inside the cells using methodology adapted from Nuovo (1995). Cell cultures were detached, rinsed, and smeared on enhanced adherence glass microscope slides (SuperFrost—Fisher). The slides were air dried and fixed for 18 h in 10% formalin neutral buffer. Digestion was performed with 2 mg/ml pepsin in 0.1 N HCl (20 min), followed by pepsin inactivation solution (100 mmol/L Tris–HCl, 100 mmol/L NaCl, pH 7.4) applied for 1 min, and were rinsed in DPBS and a final wash in absolute ethanol. Samples, run in duplicate, were surrounded with a 15 mm × 15 mm frame seal chamber (Bio-Rad, Hercules, CA, United States) for the PCR mix.

The PCR mixture was 4.0 mmol/L MgCl_2_, 0.4 mmol/L dNTPs, 1 μmol/L primers (Operon Biotechnologies, Huntsville, AL, United States), 0.06% bovine serum albumin, 8 μmol/L digoxigenin-11-dUTP (dig) (Hoffman-La Roche, Basel, Switzerland), and 0.053 U/μl of HotStart Amplitaq Gold DNA Polymerase (Applied Biosystems, Foster City, CA, United States). IS-PCR was directed to a segment of the *tax* region of the BLV genome (nt 7197–7570, F: CTTCGGGATCCATTACCTGA and R: GCTCGAAGGGGGAAAGTGAA), with an expected product of 373 bp. PCR mix was placed into the chambers of the slides and was sealed with the plastic cover of BioRad. Slides were placed into an IS-PCR machine (Hybaid Thermo OmniSlide; Cambridge Biosystems, Cambridge, United Kingdom) for amplification. Thermal profile was used as previously described (Buehring et al., 2014). After amplification, endogenous peroxidase was quenched 30 min in 3% hydrogen-peroxide solution prepared in methanol. Dig-labeled nucleotides incorporated into PCR products were detected by anti-dig antibodies in an avidin–biotin-immunoperoxidase reaction (Hoffman-La Roche) and were revealed by diaminobenzidine (DAB) solution followed by the manufacturer’s instruction (Vector, Burlingame, CA, United States). Smears of FLK cell line were used as a positive control. As a negative control of reaction, an adjacent smear for each cell line prepared without Taq polymerase and without primers was evaluated to verify that no cross-reaction or non-specific attachment occurred by the DIG-labeled uracil and/or by the anti-dig monoclonal antibody. Results were visualized in a Nikon Eclipse E200 optical microscope under × 40 magnification. Visualization of dark brown-red stain was considered a positive result. After verification of stable infection after 16 weeks, cells were stored in liquid nitrogen for further analyses.

## Results

All of the aliquots obtained from cell culture were validated for human GAPDH after 48 hpi and in each aliquot after DNA extraction for the follow-up. *Cytochrome C oxidase* of sheep was negative in all of them, confirming that it was not a cross-contamination from FLK (Supplementary Figures 2, 3).

**FIGURE 1:**
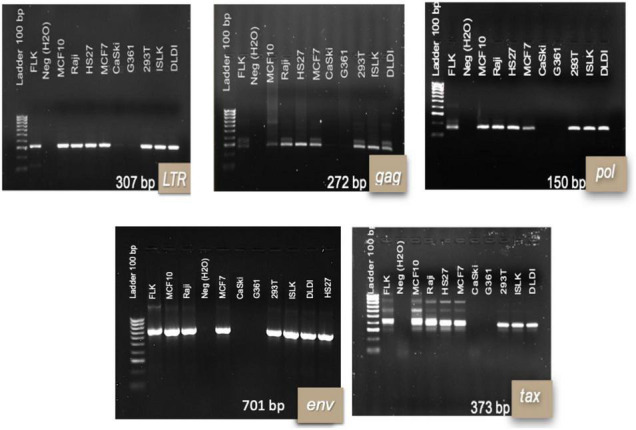
Bovine leukemia virus genes’ fragments for infected human cell lines at time 0 post-infection. 1.5% agarose electrophoresis gels with representative results per cell line. Results are shown at 48 hpi of the TransWell system. Each infection of the cell lines was repeated twice in two independent moments. DNA from FLK cell line was used as a positive control for PCR reactions.

Success of TransWell infection and stability of infection varied among different cell lines. CaSki and G361 cell lines were negative for most BLV genes following incubation in TransWells. DLDI, 293T, Raji, MCF-102A, HS-27, MCF-7, and iSLK cell lines were susceptible to BLV infection, as detected by nPCR amplification of viral genes. In time zero post-infection, all of the amplified regions for viral detection were present in 7 out of the 9 cell lines (Figure 1).

**FIGURE 2:**
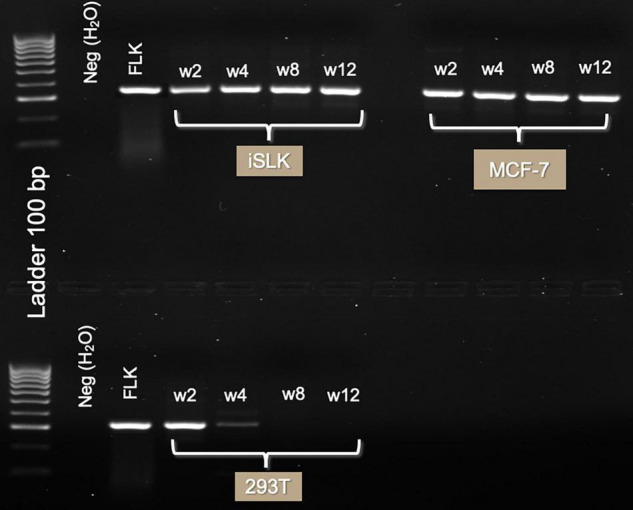
Three-month follow-up of BLV infection in human cell lines with GRE region. Gel electrophoresis of PCR product (285 bp) run on 1.5% agarose TBE gel, stained with ethidium bromide. Lanes in order are of DNA extracted 2 weeks, 1 month, 2 months, and 3 months after TransWell infection. Notice the high intensity of the bands on iSLK and MCF-7 cell lines. In 293T cell line, viral infection was lost after week 4 post-infection.

**FIGURE 3:**
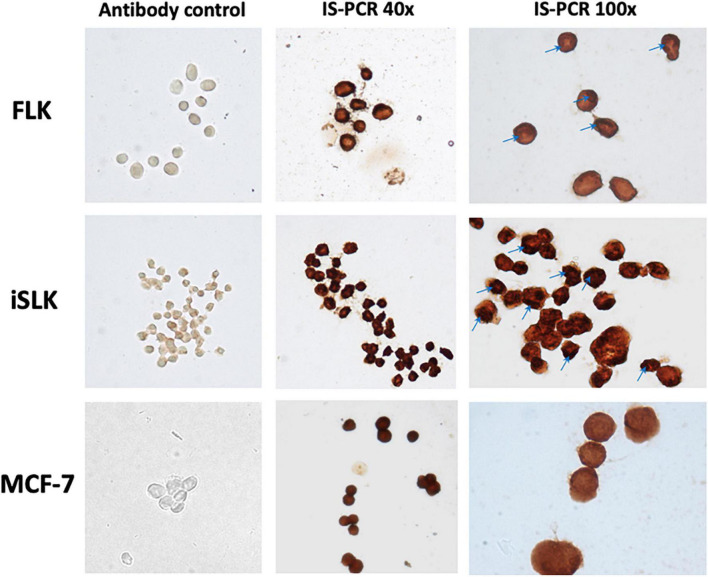
Evidence of BLV inside the cells through *in situ* PCR (*tax* region). Smear of infected cell lines. FLK—positive control cell line. iSLK-BLV positive (kidney). MCF-7 BLV positive (breast). Antibody control—*in situ* PCR without primers and TaqPolymerase control, verification of no cross-reaction of anti-dig antibody. *In situ* PCR with complete reaction. Stain is visualized by DAB reaction against anti-dig system. Images visualized at 40 × and 100 × on a Nikon Eclipse E200 optical microscope. All images were treated with the same conditions of color and contrast.

The DLDI, 293T, Raji, MCF-102A, and HS-27 cells were unable to sustain infection. In these cells, detection of BLV DNA decreased with time and became non-detectable over the follow-up period. The results of nested and *in situ* PCR are represented on Figures 2, 3, respectively, in which cell lines that reached a stable infection are described.

The presence of the GRE biomarker in the cell lines throughout the 3-month follow-up (Figure 2) is evidence of a stable infection. MCF-7 and iSLK were positive during the complete follow-up. No discernable changes in morphology of the cells infected with BLV were noted.

Figure 3 shows the results of *in situ* PCR as an end-point experiment performed on MCF-7 and iSLK after 16 weeks post-infection to visually validate the results of nPCR on cell lines with stable infection. Areas of the cells stained red/dark brown indicate regions containing BLV DNA. The cell lines iSLK and MCF7 showed BLV-*tax* gene segment inside the cells evenly distributed, with some darker spots visualized in the nucleus of iSLK (Figure 3—100 ×).

After 18 months of freezing in liquid nitrogen, cells were recovered again in cell culture to verify if they still were infected. An immunohistochemistry directed to p24 protein of the virus was carried out and detection was performed with DAB reagent in an immunoperoxidase system. Presence of the virus was confirmed in MCF-7 cells as visualized with dark coloring inside the cells. In addition, multinucleated cells were visualized suggesting the presence of syncytia formation (Supplementary Figure 4). iSLK cells were not possible to recover after thawing.

## Discussion

Understanding the impact that BLV infection could have in human beings is a topic of concern. Previous evidence of BLV in humans has reported its presence in blood, lung, and breast tissues from people with and without cancer (Mesa et al., 2013; Buehring et al., 2014; Robinson et al., 2016; Khalilian et al., 2019). This study shows that several human cell lines from different tissues were susceptible to BLV infection, and that a stable infection was obtained in two of the evaluated cell lines (MCF-7 and iSLK). Results showed the presence of viral DNA, which could be as a provirus, as total DNA was extracted for the follow-ups. Susceptibility of infection was shown in cells from different tissues such as kidney, colon, fibroblasts, and breast. These results open the possibility that BLV could be present in other organs in human beings, and considering its oncogenic potential, it will be interesting to evaluate if there is an association with other cancer etiologies in addition to breast (Buehring and Sans, 2020) and lung cancer (Kim et al., 2018).

Previous evidence of BLV *in vitro* infections has been focused on understanding specific pathways and molecular mechanisms of the virus (Inabe et al., 1998; Domenech et al., 2000; Takahashi et al., 2005; Camargos et al., 2014; Iwan et al., 2014; Murakami et al., 2017; Cuesta et al., 2019). However, few studies included human cell lines in the experimental designs (Altaner et al., 1989; Murakami et al., 2017; Suzuki et al., 2018). Results published by Altaner et al. (1989) were directed to evaluate the implications of BLV infection in humans’ cells of neurotropic origin. Establishing an *in vitro* model that will allow the research community for further studies in the biological mechanisms of BLV infection in humans is still a priority in the research field. We propose that iSLK and MCF-7 cell lines could be used in the future for further analyses.

Previously, Suzuki et al. (2018) analyzed the early stages of the *in vitro* infection of the virus, in which it was determined that one of the crucial stages for viral producing cell lines and for stable infections was the retrotransciption. As a retrovirus, once BLV enters the host cell, its RNA genome is expected to be retrotranscribed to viral DNA and remains as extrachromosomal double-stranded DNA (E-DNA). Unlike retroviruses such as HIV, translocation of the viral genome into the nucleus is weak and mainly relies on cell division processes (MacLachlan and Dubovi, 2017). However, once into the nucleus, integration in the host genome occurs mediated by viral integrases in which a proviral state is acquired, generating stable and persistent infections in the host (Grandgenett et al., 2015; Martin et al., 2016). When E-DNA remains in the cytoplasm of the cell and does not manage to be translocated into the nucleus, viral DNA is more likely to degrade inside the cells and is not possible to obtain stable infections (Reyes and Cockerell, 1996).

Our results showed that seven out of the nine cell lines were able to overpass the initial steps of the viral cycle, such as attachment, entrance, and retrotranscription. Even if it was expected a cell-to-cell infection as few amounts of free viral particles are released, independently of which was the mechanism used by the virus for the entrance in human cell lines, evidence of DNA obtained from the extractions in the follow-up indicated that initial steps were successfully fulfilled. Viral receptors proposed for BLV (AP3D1 and CAT1/SLC7A1) (Corredor et al., 2018; Bai et al., 2019) are proteins that are widely distributed in most of the eukaryotic cells, as are proteins involved in intracellular transport processes. Those proteins could be favoring the interactions between the virus and different host cells due to the high identity and similarity percentages among species (Dell’Angelica and Angelica, 2009; Fotiadis et al., 2013), and their low specificity with tissues and specific cells. For the case of CaSki and G361 cell lines, which were the only two cell lines in which the infection was not successful, it might be due to lower amounts of expression of the receptor proteins compared with other cell types (Human Protein Atlas, 2021a,b) or to the lack of capacity of inducing nanotube formation for the viral entrance (Pique and Jones, 2012; Gross and Thoma-Kress, 2016).

The cell lines in which BLV genome was detected for a longer term support the hypothesis of a stable infection in MCF-7 (breast), iSLK (kidney), and DLD-I (colon) cell lines. Even if in our study we did not measure active viral production, nor functionality of the viral genes, the identification of these cell lines with the presence of viral DNA after a long-term infection suggests that a stable infection in the human body may occur as well. Although the present study does not suggest a mechanism as to why certain cell lines were unable to maintain a stable BLV infection, we hypothesize that it could be related with the process of integration in the host genome. Considering that BLV does not have a specific profile for integration within the host genome, and that it occurs randomly in different regions, with different patterns and in several chromosomes in cattle without a specific association with disease progression (Onuma et al., 1982; Miyasaka et al., 2015), this also could be occurring in the human cells. In cattle, it is expected to integrate about 2 to 6 copies of BLV genome in the host cells, what favors to the increase of proviral load in the host due to the clonal expansion related to the disease (Gillet et al., 2007). As there is no correlation for the integration profiles in which different number of copies could be integrated, in our experiments, changes in the intensity of the bands present in the electrophoresis and darker colors in the *in situ* PCR could be associated with higher amounts of viral genome. The localization of BLV in the nucleus in iSLK *versus* the cytoplasm for MCF-7 based on the results from IS-PCR could be interpreted as differences in the integration profiles of the BLV in the form of provirus. It could be associated with more copies integrated in iSLK in contrast with MCF-7; however, in this study, we did not look for the integration sites of the virus, as it was not the main objective of the article. Finally, stable and persistent infections could also be related with the cell cycles of the different cell lines, in which initial cells that were infected with BLV could die before its mitotic cycle or could be displaced by uninfected cells replacing the cell populations.

One of the strengths of this study were the steps taken to ensure that the results obtained were free from cross-contamination. All DNA samples extracted from the human cell cultures were negative in PCR amplification of sheep cytochrome *C*, which makes it unlikely that positive nested PCR results were due to FLK cell-line contamination. Furthermore, FLK control DNA and cell line were handled in different areas, separate from the human cell culture samples to avoid cross-contamination while processing and to ensure the positive results obtained from DNA within the human cell culture samples. Two different detection techniques were used as well to ensure reliability of our results. IS-PCR visualization showed presence of the viral genome inside the cells, which could be both in the cytoplasm of the cells or in the nucleus. Even if liquid-phase PCR is unable to provide any information as to the location of the source of the DNA, it showed the presence of viral genome after a total DNA extraction, indicating that early steps of the viral cycle were performed.

In addition, more than one BLV genome region was used for the follow-up of the experiments. The LTR (long terminal repeat) promoter region of BLV was used for liquid-phase PCR, and *tax* region, which codes an auxiliary protein with oncogenic potential, was used for IS-PCR. Those regions were chosen because they are the most highly conserved regions of BLV genome (Zhao et al., 2007; Barez et al., 2015). It is thought that in BLV and HTLV, the gag–pol (polymerase)–env segment of the BLV genome is often deleted during the progression of the disease to escape the host’s immune response (Gillet et al., 2007; Zhao and Buehring, 2007). Detection of genomic biomarkers as evidence of the viral infection by PCR could result in false negatives if these regions were the primary or sole target for assays. Furthermore, it is important to highlight that both LTR region and Tax protein are crucial for active transcription of the virus within the host cells, as Tax protein acts as the main transactivator of the LTR promoter region, inducing active transcription of the virus (Fox et al., 2016; Pluta et al., 2020). In further studies, it will be interesting to determine levels of expression of both biomarkers and to identify if is there any correlation with the cell cycle, viral transcription, and cellular transformation patterns.

A limitation of our current study is that the positive results from the liquid-phase PCR do not provide information as to the integration status of BLV with respect to the host genome. While these results show that viral retro-transcription occurred (Inabe et al., 1998), we could not be sure if the virus was able to integrate its genome with that of the host cell, which is an essential step for its replication and persistent infection. This is an area of future research as inverse PCR techniques allow for determination of the integration status of the retrovirus, and can also provide information about where in the host’s genome the virus integrates, which could give an insight of its localization in the host genome and of possible cellular genes’ alteration (Murakami et al., 2011). However, inverse PCR assays are challenging as large amounts of PCR products are required for sequencing, which could be influenced by the proviral load in the host. Further studies are needed to understand the impact of the infection of BLV in these cell lines and in human beings.

Of special interest, the mammary epithelial cell line MCF-7 was both susceptible to BLV infection and able to maintain a stable infection over the 3-month culture period. Both conditions seem to be important in the progression of the disease in its host (Lairmore, 2014). This result is significant as it provides *in vitro* experimental evidence consistent with the hypothesis of BLV as an exogenous etiological agent of human breast cancer, obtained through a zoonotic infection (Corredor-Figueroa et al., 2021). Nevertheless, the varying susceptibility and capacity of maintaining a stable infection of BLV in human cell lines as well as from other mammal species opens new questions in BLV research in which further studies are needed toward the characterization of the mechanisms involved in BLV infection in other hosts than cattle. Our results provide evidence of BLV being capable of infecting human cell lines, what supports the hypothesis of natural infection of BLV in human beings, with huge possibilities of infecting different tissues among the human body.

## Conclusion

Bovine leukemia virus was able to infect human cell lines through a cell-to-cell model. BLV biomarkers were identified in the cell lines as evidence of a stable infection. iSLK and MCF-7 cell lines were those that showed positive for a longer term, even after freezing and thawing. Our results support the hypothesis of BLV being a zoonotic infection and are the basis for further studies for a better understanding of BLV mechanisms in human beings. Stable infected cell lines will be useful for future studies to be performed under controlled conditions at the laboratory level.

## Data Availability Statement

The original contributions presented in the study are included in the article/Supplementary Material, further inquiries can be directed to the corresponding author/s.

## Author Contributions

NO-G and GB: conceptualization and visualization. NO-G, KT, SB, and HS: methodology. NO-G: software. GB: validation, resources, project administration, and funding acquisition. NO-G, SB, HS, and GB: formal analysis. NO-G, MG, and GB: investigation. NO-G and SB: writing—original draft preparation. NO-G, MG, and GB: writing—review and editing. MG and GB: supervision. All authors have read and agreed to the published version of the article.

## Conflict of Interest

The authors declare that the research was conducted in the absence of any commercial or financial relationships that could be construed as a potential conflict of interest.

## Publisher’s Note

All claims expressed in this article are solely those of the authors and do not necessarily represent those of their affiliated organizations, or those of the publisher, the editors and the reviewers. Any product that may be evaluated in this article, or claim that may be made by its manufacturer, is not guaranteed or endorsed by the publisher.
