# Supplementary material for: In vitro Susceptibility of Human Cell Lines Infection by Bovine Leukemia Virus
Source: Front Microbiol. 2022 Mar 14;13:793348. doi: 10.3389/fmicb.2022.793348 (PMC8964291; doi:10.3389/fmicb.2022.793348)
Supplement: Supplementary file 1 [file Data_Sheet_1.PDF]

Supplementary Material

BLV *in vitro* infection – Olaya et. al.

1 Complementary gels, images and tables

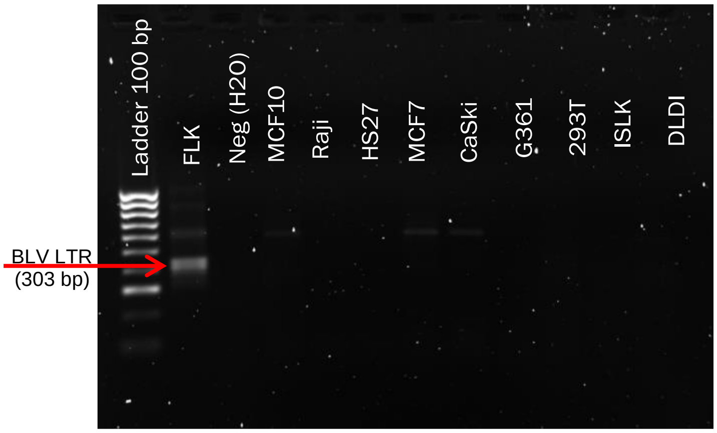

Supplementary Figure 1. Verification of cell lines before BLV infection. Notice the lack of BLV LTR region (303bp) in cell lines.

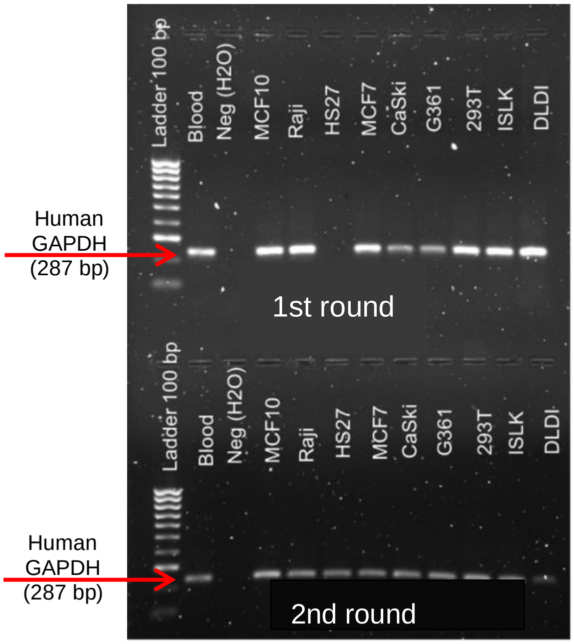

Supplementary Figure 2. Representative gel of Human GAPDH (287bp) validation after DNA extraction. *HS27 experiments were repeated for a third independent experiment, as GAPDH in experiment one was negative.

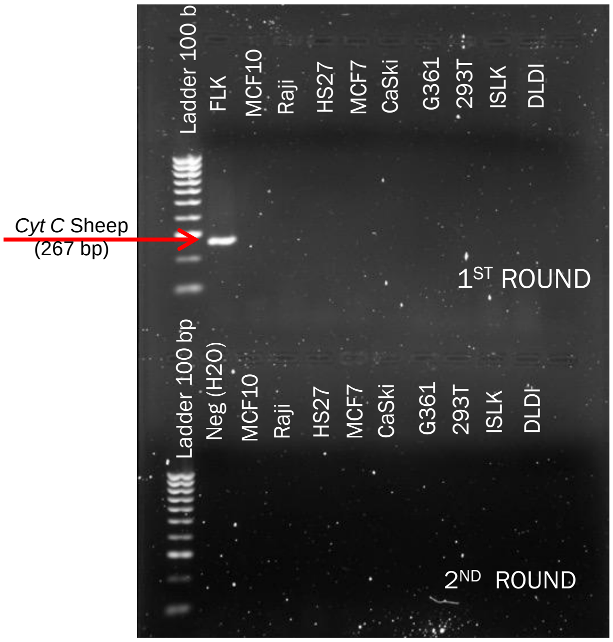

Supplementary Figure 3. Validation of Cytochrome C in cell lines after infection as lack of cross contamination with FLK

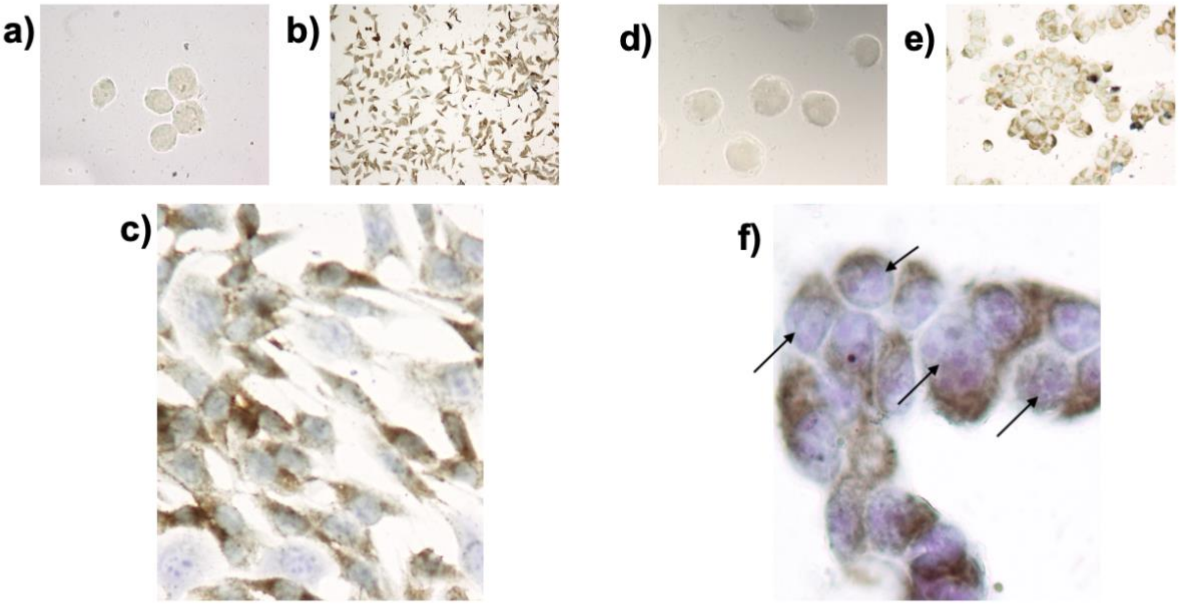

Supplementary Figure 4. Immunocytochemistry directed to p24 protein of BLV for long-term infection verification. Cells were recovered in cell culture after 18 months of being frozen. Test was revealed with DAB reagent in an avidin-biotin-immunoperoxidase assay. (a-c) FLK cells (Positive control). (d-f) MCF-7 infected cells. (a) and (d) cells smear, antibody control – reaction only with secondary antibody to confirm no cross-reaction occurred in the cells. (b) and (e) Cell culture monolayer labeled with anti-p24 antibody. Brown color indicates presence of p24 protein of the virus. Original magnification 40x. (c) and (f) Cells after immunocytochemistry stained with RAL

Diff Quick solution II (basophilic stain). Blue – purple color indicates nuclei structures. Arrows indicate presence of multinucleated MCF-7 cells. Original magnification 100x. *iSLK cell line lost viability in the freezing process and was not possible to recover in cell culture.

Supplementary Table 1. Nested PCR conditions and primers set

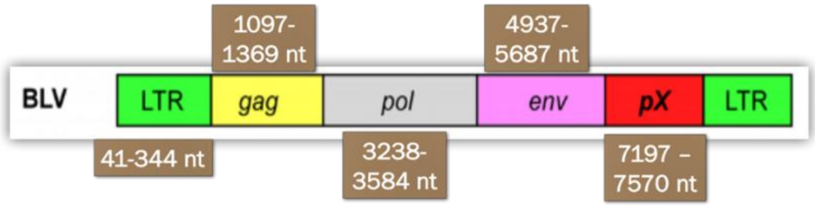

**BLV genes**	**Expected fragment**	**Primers (outer and inner PCR)**	**Annealing temperature (ºC)**	**Extension time (seconds)**
LTR-GRE	307 bp	***Outer***<br>PF - TAGGCAGCCGCCACCGC<br>PR - GCGGTGGTCTCAGCCGA<br><br>***Inner***<br>PF - CGTAAACCAGACAGAG<br>PR - TCAGCCGAGAACCAC	57<br><br>54	35<br><br>30
gag	272 bp	***Outer***<br>PF - AACACTACGACTTGCAATCC<br>PR - GGTTCCTTAGGACTCCGTCG<br><br>***Inner***<br>PF - ACCCTACTCCGGCTGACCTA<br>PR - CTTGGACGATGGTGGACCAA	54<br><br>56	28<br><br>26
env	701 bp	***Outer***<br>PF - CGGGCAAAACAATCGTCGGT<br>PR - GGAAAGTCGGGTTGAGGG<br><br>***Inner***<br>PF - CTCTCCTGGCTACTGACC<br>PR - GGAAAGTCGGGTTGAGGG	55<br><br>55	45<br><br>40
pol	157 bp	***Outer***<br>PF - TAGCCTACGTACATCTAACC<br>PR - AATCCAATTGTCTAGAGAGG<br><br>***Inner***<br>PF - GGTCCACCCTGGTACTCTTC<br>PR - TATGGGCTTGGCATACGAGC	55<br><br>55	28<br><br>36
tax	385 bp	***Outer***<br>PF - TATTTCCACCTCGGCAC<br>PR - ATTGGCATTGGTAGGGCT<br><br>***Inner***<br>PF - CTTCGGGATCCATTACCTGA<br>PR - GCTCGAAGGGGGAAAGTGAA	56<br><br>54	35<br><br>30
